# A Systematic Review of Physical Rehabilitation of Facial Palsy

**DOI:** 10.3389/fneur.2020.00222

**Published:** 2020-03-31

**Authors:** Annabelle Vaughan, Danielle Gardner, Anna Miles, Anna Copley, Rachel Wenke, Susan Coulson

**Affiliations:** ^1^Speech Pathology Service, Gold Coast University Hospital, Gold Coast, QLD, Australia; ^2^School of Health and Rehabilitation Sciences, University of Queensland, Brisbane, QLD, Australia; ^3^School of Allied Health Sciences, Griffith University, Gold Coast, QLD, Australia; ^4^Speech Science, The University of Auckland, Auckland, New Zealand; ^5^School of Health Sciences, Discipline of Physiotherapy, The University of Sydney, Camperdown, NSW, Australia

**Keywords:** central facial palsy, rehabilitation, exercise, systematic review, therapy

## Abstract

**Background:** Facial palsy is a frequent and debilitating sequela of stroke and brain injury, causing functional and aesthetic deficits as well as significant adverse effects on quality of life and well-being. Current literature reports many cases of acquired facial palsy that do not recover spontaneously, and more information is needed regarding the efficacy of physical therapies used in this population.

**Methods:** A systematic search of eight electronic databases was performed from database inception to December 2018. Gray literature searches were then performed to identify additional articles. Studies were included if they addressed physical rehabilitation interventions for adults with acquired facial palsy. Reasons for exclusion were documented. Independent data extraction, quality assessment, and risk of bias assessment followed the Preferred Reporting Items for Systematic Reviews and Meta-Analyses (PRISMA) guidelines.

**Results:** Following abstract screening, a total of 13 full-text articles were identified for independent screening by two reviewers. This included four randomized control trials, two non-randomized control trials, one cohort study, and six prospective case series studies. Twelve out of the 13 included studies reported on facial palsy as a sequela of stroke. A total of 539 participants received intervention for facial palsy across the 13 included studies. Therapy design, length and frequency of intervention varied across the studies, and a wide range of outcome measures were used. Improvement on various outcome measures was reported across all 13 studies. The quality of the evidence was low overall, and most studies were found to have high risk of bias.

**Conclusions:** All the studies in this review report improvement of facial movement or function following application of various methods of physical rehabilitation for facial palsy. Methodological limitations and heterogeneity of design affect the strength of the evidence and prevent reliable comparison between intervention methods. Strong evidence supporting physical rehabilitation was not found; well-designed rigorous research is required.

## Introduction

The facial nerve (CNVII) plays a critical role in multiple complex functions of human life including mastication, speech, and successful social communication through expression of mood and emotion (1–4). Central facial palsy (CFP) results from damage to the central segment of this nerve (facial nucleus in the pons, motor cortex, or connections between the two) (5) and manifests typically as a unilateral impairment of movement opposite to the side of the injury, with predominance in the lower face (6). In contrast, peripheral facial palsy (PFP) results from injury or damage to extratemporal segments of the facial nerve (7), for example in idiopathic “Bell's” palsy, surgery such as mastoidectomy, or inflammation such as herpes zoster (Ramsay Hunt syndrome) (8).

CFP is a frequent initial symptom in patients after stroke and other neurological injury. A study conducted by Cattaneo and Pavesi (9) found that 60% of patients with first-time ischemic cortical stroke (MCA and ACA territories) presented with CFP. Other studies of stroke populations have reported a prevalence of approximately 45% (6). It is evident from multiple searches of libraries and online evidence repositories during clinical management of CFP that most of the available literature relates to rehabilitation of peripheral facial palsy (PFP), and there is very little evidence available to guide therapists working with people suffering from CFP. Whilst systematic reviews have evaluated physical rehabilitation and other management for PFP (10–12), the different etiopathogenesis of CFP suggests that rehabilitation approaches should be specifically modified for this group (13).

Spontaneous recovery of CFP has been reported in two-thirds of people at 6 months post-stroke, with approximately one-third of patients after stroke continue to present with persisting facial palsy after 6 months (14). More recently, differing opinions are emerging in the literature regarding rates of spontaneous resolution of CFP (including associated functional and QOL deficits), with some authors noting that in the absence of rehabilitation, symptoms seem unlikely to improve (15). In their study, Volk et al. (6) reported that a high percentage of patients continued to present with CFP 3 weeks post-onset, and over 60% of these patients were discharged from sub-acute rehabilitation with deficits persisting for more than 41 days post-stroke. As the available literature suggests that CFP can persist past the initial acute phase of stroke and not resolve spontaneously, people with CFP may benefit from access to a specific rehabilitation program aimed at maximizing recovery of facial movement and function (6, 13, 16).

Facial palsy can be distressing and debilitating for those affected, causing both functional and aesthetic deficits (16). Functional deficits may be characterized by facial asymmetry and weakness of the lower half of the face, drooping of the corner of the mouth, dribbling from the corner of the mouth at rest or during oral intake, reduced masticatory force and efficiency, asymmetrical smile and dysarthria (slurring or reduced clarity of speech) (15). It is well-recognized in the literature that in addition to functional deficits, facial palsy has a negative effect on quality of life (QOL) and emotional well-being (7, 17–21). In their 2016 study comparing QOL between individuals with pure CFP post stroke vs. pure dysarthria, Chang et al. (21) found that the CFP group had significantly worse scores on QOL and depression scales. Interestingly, it has been found that the presence of facial palsy alone regardless of its severity has a detrimental effect on the psychological well-being of those who experience it (19).

### Rationale

Currently, there is minimal evidence available to guide clinical decision-making in the rehabilitation of CFP (22, 23) and very little information available regarding the effectiveness of popular intervention techniques (21, 23). As mentioned above, CFP may not resolve spontaneously and the negative impacts of CFP on people who experience this disorder can be wide-ranging. Rehabilitation may maximize functional recovery and improve the quality of life and psychological well-being of people with CFP (6, 13, 16) however there is currently no comprehensive or systematic review of the literature specific to this disorder to inform therapy planning and provision. This has significant implications for patient management, as it is still not clear to health professionals whether physical rehabilitation techniques work, or which technique is most effective.

### Objective

The purpose of this review is to identify and examine the available literature specifically relating to physical rehabilitation of CFP. This review aims to (1) identify the types of physical rehabilitation methods used in remediation of CFP, (2) review the effectiveness of various methods of physical rehabilitation, and (3) review the methodological quality of the studies retrieved. The findings will be pertinent to clinicians working with patients with CFP as this is the only review that the authors are aware of that systematically evaluates the evidence base for rehabilitation of this disorder.

### Research Question

What is the effectiveness of physical rehabilitation for acquired central facial palsy in adults?

## Methods

### Study Design and Search Strategy

This review follows the Preferred Reporting Items for Systematic Reviews and Meta-Analyses (PRISMA) statement. The review protocol is registered on PROSPERO (CRD42018115303). A systematic search strategy was devised in conjunction with a senior librarian, using the core concepts of facial paralysis, central nervous system disease, and physical rehabilitation. The Medical Subject Headings (MeSH) database was used to obtain terms that were related to these concepts to ensure a comprehensive search of the literature was performed. The search strategy was designed and performed using Medline (Ovid) terminology (see Appendix 1). No limitations were used for year published, language, or publication type. The search strategy was then translated for searching the following databases: Embase (Elsevier), CINAHL (Ebsco), Cochrane Central Register of Controlled Trials, Proquest Dissertations and Theses Global, PEDro, Speechbite, and Web of Science (Clarivate).

Gray literature searches included searches of WHO ICTRP (3) and ANZCTR (0) using the terms central facial pa^*^, with no completed studies (3 currently registered trials) retrieved. ClinicalTrials.gov was searched using the heading *facial palsy* with 18 completed studies retrieved, however all retrieved studies either pertained to peripheral facial palsy or did not have results available and were therefore not included in this review. Clinical practice guidelines and best practice statements were searched for relevant literature/references, including Clinical Guidelines for Stroke Management 2017 (24), United Kingdom National Clinical Guideline for Stroke (25), and American Speech and Hearing Association Evidence Maps (https://www.asha.org/MapLanding.aspx?id=8589947062).

Further hand-searching of library and clinical databases were conducted. Specialists from facial therapy services in Australia and internationally were asked to provide any relevant literature which informs their current clinical practice. The reference lists of articles eligible for inclusion following full text screening were searched, and any titles that appeared to fit the criteria set were retrieved.

### Participants, Interventions, Comparators

The inclusion and exclusion criteria for the review are presented in Table 1.

**Table 1:** Selection criteria.

**Selection Criteria**	**Inclusion Criteria**	**Exclusion Criteria**
Participant	Adults with acquired CFP	Pediatrics (<18 yrs)
Intervention	Physical rehabilitation of CFP	Surgical or pharmacological intervention with no physical rehabilitation component
Comparator	None or placebo treatment, drug/surgical treatment, or other physical rehabilitation	No outcomes reported
Outcomes	Quantitative or qualitative outcomes in subjective or objective measures of motor function or symmetry/appearance or QOL	
Other: Methodology	Case series Separate data for CFP and PFP	Single case study design, secondary research (i.e., reviews) Combined data for CFP and PFP or unclear delineation
Other: Publication details	Articles from research journals Articles in English	Book chapters, thesis publications, opinion pieces Articles not in English

### Systematic Review Protocol

**Figure 1:**
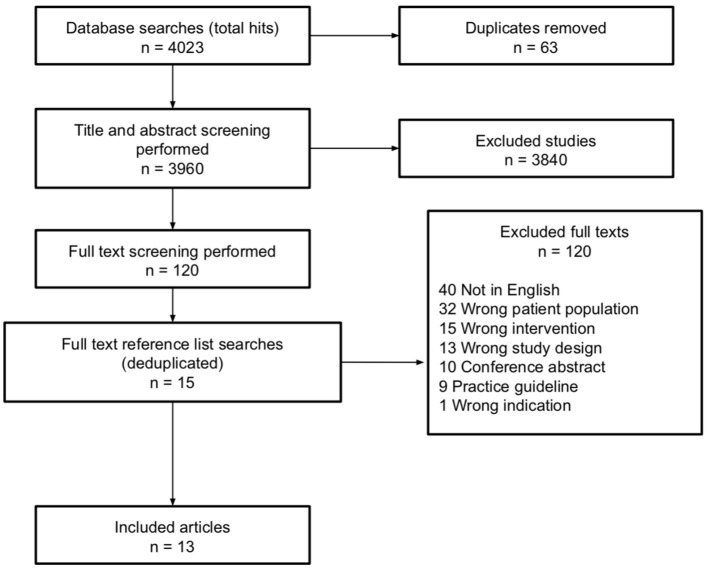
Preferred reporting items systematic reviews and meta-analyses (PRISMA) flow diagram detailing search strategy and selection criteria.

The systematic search strategy is presented in Figure 1. A senior health service librarian performed database searching. Articles retrieved in the database searches were deduplicated using the Bond University CREB SRA deduplicating tool (http://crebp-sra.com) and then further screened to remove other duplicates. Abstracts of all articles remaining following deduplication were then collated into an Endnote library, which was then uploaded to Covidence (Veritas Health Innovation Ltd, Melbourne, Australia) for blind review by two independent reviewers (AV and DG). Titles and abstracts were screened against the predetermined inclusion/exclusion criteria and subsequently added to full text screening lists. Articles included by both independent reviewers and articles that were marked as “maybe” by one or both reviewers were considered eligible for further review. Full texts of eligible studies were then retrieved and independently assessed for inclusion/exclusion. Any conflicts that arose during eligibility assessment were resolved by (a) discussion between reviewers, or where agreement could not be reached, by (b) discussion with the review team and relevant experts in the field.

### Data Extraction

For all included articles, a range of variables including study population/participant details, selection criteria, methodology, interventions (therapy approach, intensity, follow-up) and outcomes were extracted and are presented in a descriptive summary in Table 2. These variables were identified as most relevant to our clinical question. Data extraction was performed initially by the second author (DG), and then amended and expanded where necessary by the first author (AV) using Google Sheets (Google, CA, USA). Due to heterogeneity in the included studies a meta-analysis was not able to be performed.

**Table 2:** Extracted data.

**Study ID [References]**	**Participants N Dx time post onset**	**Methodology**	**Interventions**			**Intensity length of Tx freq duration**	**Outcome measures**	**Results**
				**Group A**	**Group B**			
Hagg and Anniko (26)	30 (24 with UFP) Stroke 2 days−10yrs	Retrospective case series	Active Therapy	Lip muscle training	N/A	>5 weeks 3 × 3/day 5–10 s	Swallowing capacity (ml/s) Lip force	Stat sig improvement in both OMs (*p <* 0.05) FP 'improved'
Hee-Su et al. (27)	10 Stroke <6 mths	Prospective case series		Traditional therapy + resistance training of OO	N/A	4 weeks 5 × /wk *not stated*	Orbicularis oris strength Lip closure (VDS)	Stat sig improvement in both OMs (*p <* 0.05)
Huffman (28)	4 Brain injury >2 mths	Prospective non-randomized control trial		Mirror therapy	Mirror therapy + EMG	10 days (in 2 week period) Daily 30min	Muscle grade	Improvement in both pairs although 3 × greater in EMG vs. mirror
Kang et al. (23)	21 Stroke <12 wks	Prospective RCT		Orofacial exercises	Orofacial exercises + mirror therapy	14 days 2 × /day 15 min	HBGS Facial movement difference (m-dif) Facial movement ratio (m-rat)	Stat sig improvement in all OMs (*p <* 0.05), greater in mirror vs. control
Choi (29)	9 Stroke <3 mths	Prospective case series	Passive Therapy	Neuromuscular ES + dysphagia therapy	N/A	4 weeks 5 × /wk 30 min/session	Max cheek strength (MCS) Max lip strength (MLS) Dysphagia (VDS)	Stat sig improvement in MCS and MLS Stat sig decrease on VDS (*p <* 0.05)
Konecny et al. (16)	99 Stroke 1-2wks	Prospective RCT		SSRI, SP/OT/PT	SSRI, SP/OT/PT + orofacial therapy	4 weeks Daily not stated	HBGS Distance measure BDI-II	Stat sig improvement in all OMs (*p <* 0.05), greater in experimental vs. control
Konecny et al. (13)	99 Stroke 1–2wks	Prospective RCT		SSRI, SP/OT/PT	SSRI, SP/OT/PT + orofacial therapy	4 weeks Daily not stated	As above + Bartel index Mod. Rankin score	Stat sig improvement in both QOL OMs (*p <* 0.05), greater in experimental vs. control
Zhou and Zhang (30)	165 Stroke 1day−6yrs	Prospective RCT		Scalp acupuncture	Western medicine	24 days? Daily ~50 min	Clinical indexes / function grading scales	Improvement in 88.57% of acupuncture group and 76.67% of western medicine group
Hagg and Larsson (31)	7 Stroke 6 mths−4 yrs	Prospective case series	Combination Therapy	Body regulation, manual orofacial regulation, palatal plate activation + velopharyngeal closure training	N/A	5 weeks 5 × /wk 120 min/session + HEP	Swallowing capacity (ml/s) Meal observation Oral motor performance Orofacial sensory function Velopharyngeal closure VFSS Self-assessment	Improvement on raw scores in at least one variable for all OMs
Hagg Tibbling (32)	31 Stroke Days−10 yrs	Prospective non-randomized control trial		Palatal Plate (PP)	Oral IQoroR screen (IQS)	3 months 3 × /day PP 10–30 min; IQS 30 s	Swallowing capacity (ml/s) Facial Activity Testing (FAT)	Stat sig improvement in both OMs for both groups (*p <* 0.05) Improvements maintained at 1 yr f/up
Noor et al. (33)	50 Stroke *Not stated*	Prospective case series		Massage, ES, KOBAT	N/A	?3 weeks 3 × /wk *not stated*	Spasticity grade	Reduction in spasticity grade for all participants
Van Gelder et al. (34)	2 Stroke 2mths	Prospective case series		Neuro Developmental Treatment	N/A	9–12 weeks Weekly *not stated*	Mimic expressions Orofacial function Asymmetry and adequacy	2/2 improved mimic expressions + symmetry 1/2 improved orofacial function + adequacy
Volk et al. (6)	112 Stroke 20 days (median)	Prospective cohort study		Physical training of related muscles, tapping, mirror therapy	N/A	21 days (median) *not stated not stated*	Bartel index HBGS Sunnybrook FGS Stennert index Action units (AU) FaCE questionnaire FDI	Stat sig improvement in activity, facial nerve motor function, self-reported non/motor abilities (*p <* 0.05)

*Dx, Diagnosis; EMG, Electromyography; ES, Electrical stimulation; HBGS, House Brackmann Grading Scale; FGS, Facial Grading System; FDI, Facial Disability Index; FP, Facial palsy; HEP, Home exercise program; OM, Outcome measures; OO, Orbicularis oris; OT, Occupational therapist; PT, Physiotherapist; QOL, Quality of life; SP, Speech pathologist; SSRI, Selective serotonin reuptake inhibitor; Tx, Therapy; UFP, Unilateral facial palsy; VDS VFSS, Dysphagia Scale; VFSS, Videofluroscopic Swallow Study*.

### Quality Assessment

Risk of bias was assessed using tools appropriate to the study methodology determined during the data extraction process: case series reports were assessed using the JBI Critical Appraisal Checklist for Case Series (35), cohort studies were assessed using the JBI Critical Appraisal Checklist for Cohort Studies (35), and control trials were assessed using the Physiotherapy Evidence Database—Psychbite scale (PEDro-P) (36). No mixed-method studies were identified during the search, therefore the tools used to assess risk of bias were altered from the original PROSPERO protocol to be more appropriate to the various study designs retrieved (control trials, case series, cohort study). Risk of bias analyses were performed independently by two reviewers (AV and RW) and discrepancies were discussed by the two authors until consensus was achieved.

### Data Analysis

Meta-analysis of the results was not indicated due to the clinical diversity of the studies retrieved, as recommended by the Cochrane Handbook for Systematic Reviews of Interventions (37). Each of the studies retrieved described differing experimental designs, treatment protocols, and methods of outcome measurement, and there was inadequate reporting of data and statistics necessary for appropriate and meaningful meta-analysis. Types of physical rehabilitation used in CFP have been broadly grouped as having used either an active approach (recipients actively move their own muscles or structures to perform exercises or volitional muscle movements), passive approach (movement is facilitated by external force, person or device e.g., massage/stretching, acupuncture, electrical stimulation), or a combination of the two. The effectiveness of physical rehabilitation has been determined by the examination of the reported results in those studies that provided sufficient data and is discussed in the context of the various grouped approaches. Rating of the overall quality of the evidence has been performed by applying relevant sections of the Grades of Recommendation, Assessment, Development, and Evaluation (GRADE) approach to individual studies (38).

## Results

### Study Selection

The results from database searching and selection processes are shown in Figure 1.

### Study Characteristics

Six case series (total no. of subjects = 108) one cohort study (total no. of subjects = 112), and six control trials (total no. of subjects = 133) were identified. Methodological details are outlined in Table 2. Of the four RCTs, two appeared to use identical participant populations and outcome data and are subsequently discussed as one study in parts of this review (*n* = 99 (13, 16). All the included studies used a pre-test post-test design.

### Participant Demographics

Participant demographics for all included studies are reported in Table 2. Twelve out of the 13 included studies reported on facial palsy as a sequela of stroke, and one study reported facial palsy secondary to acquired brain injury. A total of 539 participants received intervention for facial palsy in the 13 studies included in this review (age range 48–88 yr old). There was a large range in time post-onset of facial palsy from acute (e.g., “days”) to chronic (e.g., 6–10 years) stages of recovery.

### Types of Physical Rehabilitation for Facial Palsy

There was a high degree of heterogeneity in physical rehabilitation methods described for adults with CFP. Seven studies reported interventions aimed at remediation of facial palsy as their primary objective (6, 26–29, 31, 33), and six reported targeting lip function or movement in the context of post-stroke dysphagia therapy (13, 16, 23, 30, 32, 34). Four studies reported on active intervention methods for remediation of oromotor function or facial palsy (23, 26–28); two used muscle strengthening exercises alone (26, 27) and the other two used biofeedback (via mirror or device) while performing orofacial exercises (23, 28). Four studies reported on passive intervention techniques such as massage, stretching or electrical stimulation for the remediation of facial muscle strength or facial palsy (13, 16, 29, 30). Acupuncture is classified in this review as passive rehabilitation; one study (30) reported on the use of scalp acupuncture compared to a group that received “western medicine.” Five studies combined active and passive approaches in the rehabilitation of CFP (6, 31–34); therapy varied across these studies but all included elements of active exercise, massage, stretching or passive manipulation, or application of various devices (Table 2).

### Dosage

Length and frequency of therapy varied across the studies, with participants receiving multiple therapy sessions per week for between 10 days and 9 weeks. Details relating to intensity of therapy (length, frequency, and duration of intervention) are presented in Table 2.

### Outcome Measures

A wide range of outcome measures for muscle strength and facial movements were used including measures of muscle strength, facial movement, and symmetry; details are outlined in Table 2. No validated outcome measurement tools were used. The majority of studies did not provide detailed descriptions of grading scales; only three studies (6, 13, 16, 23) used well-known outcome measures specific to facial palsy. Facial palsy was often measured in conjunction with other deficits of speech, swallowing, emotional and psychological well-being.

### Effectiveness of Physical Rehabilitation of Facial Palsy

Four RCTs and nine observational studies reported improvements in various measures of facial palsy or facial motor function, which are outlined below in the context of the rehabilitation approach used (active, passive or combination). Eight of the 13 studies included comments about the statistical significance of the results (*p*-values), however none performed calculations of effect size, and therefore none of the studies provided sufficient data to assess imprecision or inconsistency as outlined in the GRADE approach. There were also insufficient data reported to facilitate judgement of indirectness; the nine observational studies do not undertake comparison with an alternative therapy or control group, and none of the RCTs provided calculations of risk ratio or effect size that would enable meaningful direct comparison.

#### Active Therapy

Four studies reported on active therapy methods; one RCT (23), one nRCT (28), and two case series' (26, 27). All four studies reported improvements in treatment variables measured. Kang et al. (23) reported improvement in HBGS scores and functional measures (facial movement ratios) in both the control group and the experimental group (both groups performed the exercise protocol with the experimental group receiving mirror feedback as the experimental condition). Huffman (28) also reported improvement in all subjects on an unvalidated ‘muscle grade' rating scale mentioned but not detailed by the authors; as well as improvements three times greater for the subjects receiving EMG feedback compared to mirror feedback. Both the case series' implemented protocols of lip strengthening using instrument-based exercise. Hee-su et al. (27) reported improvements in orbicularis oris muscle strength and lip closure function during swallowing; no outcomes specific to facial palsy (e.g., measures of movement or symmetry) were used. Hagg and Anniko (26) also reported improvement in raw scores of lip force from baseline measures taken using a Lip Force Meter instrument however did not specifically report on outcomes for facial palsy.

#### Passive Therapy

Four studies reported on passive therapy methods, including a case series study (29) and three RCTs (13, 16, 30); two RCTs are discussed together (13, 16) for reasons mentioned previously. All four reported improvements in relevant measures. Choi (29) reported changes in facial muscle strength compared to baseline measures however did not explicitly report outcomes for facial palsy. Zhou and Zhang (30) reported a larger change in all outcome measures (including a facial movement grading scale not described in the study) for the group receiving acupuncture compared to those receiving “western medicine.” There was no detail provided regarding the method for administration of this grading scale. Konecny et al. (13, 16) reported improvements in formal facial nerve assessment measures (HBGS) as well as in a variety of other functional and quality-of-life scales.

#### Combination Therapy

Five studies reported on therapy protocols that combined passive and active methods (e.g., massage/manipulation with active exercise regime). These included three case series' (31, 33, 34), one non-randomized control trial (32) and one cohort study (6). One case series (33) reported improvements in spasticity of facial muscles; this was demonstrated by reporting the number of participants per scoring level (grade I–V) pre and post treatment on an unnamed grading tool. There were no individual assessment outcomes reported and there was an absence of statistical analysis of the data. One (31) reported improvements in raw scores of orofacial motility on an informal four-point scale as well as improvement in mean severity score of oral motor performance. The authors provided raw pre and post assessment data for each participant as rated by multiple assessors; there was an absence of further analysis of this data and overall outcomes were focused on dysphagia rather than facial palsy. The case series reported by Volk (6) reported improvements in three well-known tools to assess facial palsy [HBGS (39), Sunnybrook Grading Scale (40), and Stennert Index (41)], two validated quality of life instruments [FaCE Questionnaire (42) and FDI (43)], and a system of automated facial movement analysis described in the study.

### Maintenance of Therapeutic Effects

Eleven of the 13 included studies did not report any follow up assessment, and therefore no evaluation of the maintenance of therapeutic effects was available. One study (32) reported maintenance of improved facial activity at follow-up assessment at least 1 year post treatment in both groups. Van Gelder et al. (34) reported on follow-up assessment 9 weeks post treatment in only one of the two participants. Their results showed a decline in function between completion of treatment and re-assessment, which the authors interpreted as showing treatment effects were not maintained.

### Methodological Quality and Risk of Bias

A summary of the consensus ratings for methodological assessment is shown using modified harvest plots, which have been used previously in systematic reviews to present data that is not able to be graphed using traditional methods (44, 45). These modified harvest plots were created by grouping similar criteria together for each appraisal tool, as detailed in Table 3. As in previous studies where modified harvest plots have been used, methodological quality is represented by bar height (45). “Unclear” consensus ratings have been scored as zero when calculating scores for each criterion on the JBI tools.

**Table 3:** PEDro-P and JBI ratings.

**Condensed category**	**PEDro-P Item**	**Condensed category**	**JBI item (Case series)**	**Condensed category**	**JBI item (Cohort)**
Participant	Eligibility criteria specified Concealed allocation	Participant	Eligibility criteria specified Standard, reliable measurement of condition Valid identification of condition	Participant	Both groups similar, recruited from same population
Intervention	Prognostic similarity at baseline between intervention groups	Design	Consecutive inclusion Complete inclusion Participant demographics Participant clinical information Outcomes or follow-up	Design	Exposures measured similarly Standard, reliable measurement of exposure Confounding factors identified Groups/participants free of outcome initially Outcome measurement valid and reliable
Blinding	Subject blinding Therapist blinding Assessor blinding				
Outcomes	>85% of the subjects followed up for at least 1 key outcome Intention-to-treat analysis Between group statistical analysis for at least 1 key outcome	Site	Site demographics	Follow-up	Follow up sufficient and reported Complete follow up Incomplete follow up managed
Variability	Point estimates of variability provided for at least 1 key outcome	Statistics	Appropriate statistical analysis	Statistics	Appropriate statistical analysis

#### Control Trials

**Figure 2:**
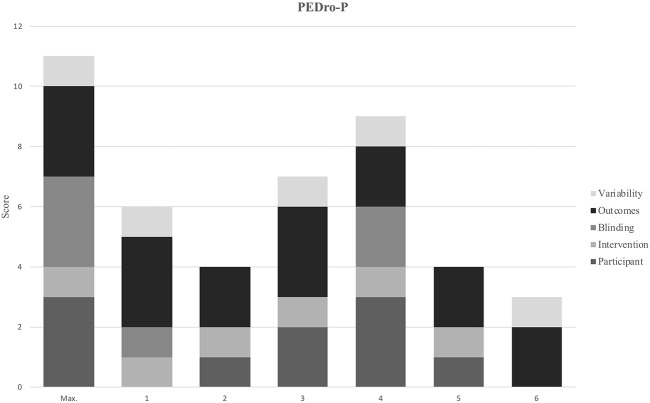
Study identification number: 1. (32) 2. (28) 3. (23) 4. (16) 5. (13) 6. (30) Max. indicates the highest possible score that an article could receive in each category.

Across the control trials, scores on the PEDRO-P ranged from 3 to 9 with an average of 5.5 out of 11 (see Figure 2). Of the RCTs, 2 of the 4 specified eligibility criteria for inclusion in the study, and while the majority allocated subjects randomly to interventions only one concealed this allocation. Blinding was an area of significant risk across the RCTs, with 1 of 4 studies blinding subjects and assessors and no blinding of therapists in any study. The nRCTs showed similar shortcomings in allocation and blinding items. The intervention groups were similar at baseline regarding the most important prognostic indicators in >90% of the studies. Outcome measurement was an area of strength for all the control studies; 100% obtained measures of at least one key outcome from >85% of subjects and demonstrated that all subjects for whom outcome measures were available received the treatment or control condition. Overall the quality of the control trials is low due to the significant limitations present in the majority of studies.

#### Case Series and Cohort Study

**Figure 3:**
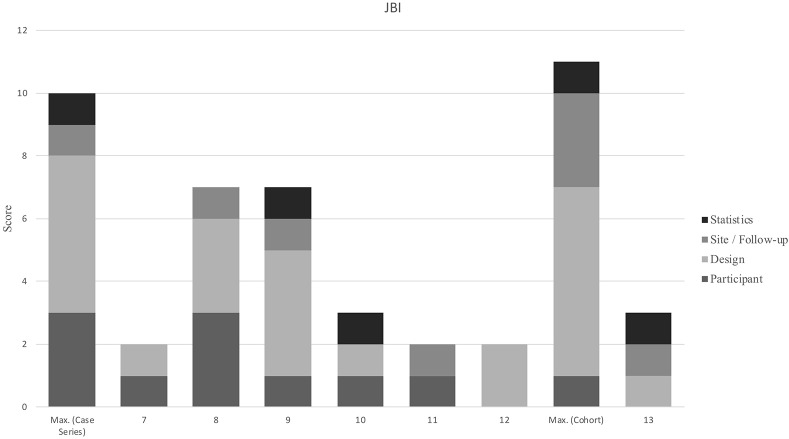
Study identification number: 7. (29) 8. (28) 9. (26) 10. (27) 11. (33) 12. (34) 13. (6) Max. indicates the highest possible score that an article could receive in each category.

Scores on the JBI tool for case series evaluation ranged from 2 to 7 with an average of 3.8 out of 10 (see Figure 3). Four (27, 29, 33, 34) of the six case studies were judged to be at high risk of bias; 2 of the 6 of studies failed to outline clear criteria, only 30% used valid methods for identification of the condition, >80% did not report consecutive recruitment of subjects and failed to clearly report clinical information of the participants. Two case studies were judged to have an unclear risk of bias (26, 31); strengths of both these studies were found in reliable condition measurement and clear reporting of participant demographics. Limitations of the case series' judged as “unclear risk of bias” were varied—in one (31) it was not clear if the study included consecutive and complete inclusion and methods of statistical analysis were ambiguous; in the other (26) criteria were not clearly defined and it was not possible to determine if valid methods for identification of the condition were used.

## Discussion

This review has shown that despite trends demonstrating improvement in CFP following various types of physical rehabilitation, there is a lack of high-quality evidence currently available to inform clinical practice. Many questions remain for clinicians planning and providing therapy for this population. Literature is emerging which recognizes CFP as an impairment in its own right (separate to its impact on speech or swallowing function), and therapies specifically targeting this disorder are being investigated with increasing frequency. All the studies in this review report improvement on various parameters of facial movement or function and, although lacking in rigor, indicate potential benefit from using physical rehabilitation approaches.

### Effectiveness of Therapy

Improvement in facial muscle strength or movement was reported across all 13 studies, and positive changes in other outcome measures such as swallow function and quality of life were also shown (where measured). Only one study provided data for maintained effects of therapy at >1 year post intervention (32), and while this study had comparative strengths in methodology, its lack of overall rigor reduced the strength of the data. All 13 studies reported statistically significant improvements from baseline measures, however none performed calculations of optimal sample size or treatment effect measures. It is therefore unclear if the improvements reported can be attributed to the physical therapy provided or if other variables influenced the outcomes. Overall, the studies included in this review provide insufficient evidence to draw strong conclusions regarding the effectiveness of physical rehabilitation for CFP.

Comparison of the effectiveness of differing therapy approaches remains unclear following this review. Of the four studies that were found to be the most methodologically sound (as per risk of bias assessments), two provided active therapy (one involved strengthening exercises only) (23, 26), one provided passive therapy (16), and one described a combination of these two approaches (32). Active and passive approaches were explored by only a small number of methodologically weak RCTs, and there were no RCT designs that investigated a combination of approaches. As well as insufficient reporting of treatment effect size or precision, there was a large amount of variation in all aspects of the design of the studies—each study described different participant variables (e.g., time post onset), dosage and treatment duration. These factors restrict any meaningful comparison being made between outcomes, which leads to a lack of support for one method of rehabilitation over another.

This review highlights the need for further well-designed and rigorous research to examine the efficacy of physical rehabilitation of CFP. The trend of improvement across various outcome measures reported in all studies provides some indication that physical therapy may be of benefit, however overall there were significant limitations that impact on confident application of these findings to current clinical practice. These include a lack of comprehensive reporting and analysis of data in all studies, and methodological limitations (e.g., lack of RCT designs, lack of concealed allocation, minimal use of blinding, and lack of follow-up assessment). The majority of studies also failed to use standardized, reliable outcome measurements, which creates questions about the validity of findings and makes any comparison of outcomes difficult. Future studies should aim for more rigor in their design, for example by using RCTs to minimize risk of bias and strengthen the validity of findings and including follow-up assessment to measure maintenance of therapeutic effect.

### Assessment

A major challenge for evaluation of methods of physical rehabilitation of CFP is the heterogeneity of assessment tools (and subsequently outcome measures) described. Only four of the 13 included studies utilized any standardized method of assessing facial palsy (6, 13, 16, 23, 32) and only three of these used widely-accepted quantitative outcome measures (6, 13, 16, 23). The remainder of the studies described a variety of informal clinical measures of muscle strength or facial movement and function. As such, comparison of findings between the studies in an effort to establish which methods were more effective is not able to be reliably performed.

### Clinical Implications

Positive trends in favor of physical rehabilitation were found. All the studies retrieved by this review process do appear to show improvements in facial palsy with rehabilitation, which lends support to the rationale for continuation of therapy provision as well as ongoing research. The strength of the evidence is low overall, which should be considered when planning intervention for this population.

### Future Directions

Future studies should aim to use objective and standardized assessment tools. Objective assessment of facial palsy is notoriously difficult (42, 46). Due to the lack of published, validated assessment tools available specific to CFP, further validation of tools designed for broader use (including peripheral types of facial palsy) may be indicated. Literature specific to PFP recommends use of the Sunnybrook Facial Grading System (40), and the House-Brackmann Grading Scale (39), and these tools have been used with some effectiveness to measure CFP in studies by Volk et al. (6), Kang et al. (23), and Konecny et al. (13, 16). There are limitations in both tools including subjective ordinal grading systems with limited items (47, 48). The Electronic Facial Paralysis Assessment (eFACE) was developed to provide clinicians with a tool that “has greater sensitivity and objectivity when assessing incomplete paralysis and post-interventional improvement…in cases of both acute peripheral nerve palsy and recovery” (49). This tool has been found to have high test-retest reliability (50), have high validity and reliability (49), and had positive feedback from a panel of international facial nerve experts (51). The tool needs further validation in a CFP population. In addition to measuring facial function, the inclusion of reliable outcome measures that evaluate the emotional and psychological impact of CFP would enable a broader assessment of the holistic impacts of rehabilitation. Two examples of validated patient-graded tools that are referenced in current CFP literature are the Facial Clinometric Evaluation (FaCE Scale) (42) and the Facial Disability Index (FDI) (43). Studies of CFP should include use of one of these tools, as non-motor impacts of facial palsy have been shown to be as important as motor function to people with this impairment (52).

It would be beneficial to have a comprehensive picture of current clinical practice to incorporate into future studies. Clinical physiotherapists and speech pathologists provide rehabilitation for CFP for using principles derived from peripheral nerve damage literature due to the lack of studies specific to CFP, despite these therapies also having low quality supporting evidence (12) and varying significantly in mechanism of impairment. A comprehensive survey of current practice would enable “expert opinion” to be integrated into the development of a gold standard of evidence-based physical rehabilitation, along with stronger evidence from well-designed clinical trials.

Exercise-based physical rehabilitation for facial palsy must be performed in a controlled and precise manner, and repeated sufficient times to induce long-term synaptic change (53). These exercises are often performed using some method of biofeedback (e.g., mirror); primarily relying on the visual system to obtain accurate proprioceptive information about position of facial muscles during slow, controlled movements that focus on symmetry (54). Without some form of external proprioceptive feedback, it is extremely difficult for patients to precisely and effectively judge and monitor the movements of facial structures (54). Exercise protocols can therefore be difficult for people to perform accurately if they have concomitant visual-perceptual, cognitive or behavioral changes secondary to stroke. Well-designed research which evaluates the effectiveness of interventions which are accessible to a wider clinical population would be of great benefit to people suffering from central facial palsy whose other impairments prevent them from engaging in strict exercise-based protocols. Regardless of the intervention strategy employed, clear and detailed reporting should be ensured to enable replicable therapeutic protocols.

Further investigation of physical rehabilitation methods for CFP is required to determine effective types and approaches for therapy and to guide clinical decision-making. There is a gap in services currently available for people wishing to access therapy for CFP and is not possible to base a strong case for clinical input on the current literature, even though trends have been identified that indicate potential benefit of physical rehabilitation.

## Limitations

Although every effort was made to ensure database and other searches were comprehensive it is possible that some records were not retrieved via the search methods. Due to the difficulties and cost associated with obtaining verified and reliable document translation, this review was unable to include articles where the full text was not available in English. This may have resulted in some studies being missed; the authors are aware of at least one non-English study (14) which may have contributed toward this review. Our systematic review also had limitations relating to methodological quality and available data in the existing literature; only four RCTs were retrieved, which were of low quality, and the observational studies all lacked sufficient data to draw strong conclusions or perform calculation of treatment effect size. It is recognized that in many areas of health care, some interventions are supported by evidence from RCTs and others are not (55). It is also acknowledged in medical research literature that decision-making is often necessary even when there is imperfect evidence (56). As clinicians who provide assessment and therapy to patients with central facial palsy, we included the smaller observational studies due to a lack of larger or more well-designed trials—as per Balshem et al. “in the absence of high-quality evidence, clinicians must look to lower quality evidence to guide their decisions” (57). While we are aware that the limitations in methodology affect the reliability of these studies, and thus also affect the strength of recommendations that can be drawn from their findings, the reality is that there are not enough large well-designed RCTs available to rely solely on this level of evidence for clinical decision-making and intervention.

## Conclusions

The studies in this review report improvement of facial movement or function following application of various methods of physical rehabilitation for CFP. Methodological limitations and heterogeneity of design affect the strength of the evidence and prevent reliable comparison between intervention methods. Strong conclusions regarding the effectiveness of intervention cannot be drawn using the studies identified by this review as good quality, robust evidence supporting physical rehabilitation of central facial palsy was not found.

## Data Availability Statement

The datasets generated for this study are available on request to the corresponding author.

## Author Contributions

AV and DG conceived the idea for this review. AV, DG, RW, AM, and SC formulated the question for review and designed the search strategy. AV and DG performed the abstract screening, full text review, and extracted data from included studies. AV and RW performed the risk of bias assessments. AV analyzed and interpreted the data and drafted the manuscript. AC and AM provided overall supervision of the project and final approval of the version to be published. All authors provided critical feedback and helped shape the research, analysis, and manuscript.

### Conflict of Interest

The authors declare that the research was conducted in the absence of any commercial or financial relationships that could be construed as a potential conflict of interest.

## Appendix

### Search Strategy (Example)

Database: Ovid MEDLINE(R) ALL <1946 to December 31, 2018>

— — — — — — — — — — — — — — — — — — — –

1. exp Facial Paralysis/ (11721)
2. ((facial or orofacial or oro-facial) adj3 (paralys* or paresis or droop* or palsy or asymmetr* or impair*)).tw. (14536)
3. ((facial or orofacial or oro-facial) adj3 (express* or nerve* or muscle* or move* or reanimat*)).tw. (26746)
4. or/1-3 (38981)
5. exp Physical Therapy Modalities/ (140199)
6. (exercis* or therap* or physiotherap* or rehabilit* or retrain* or train* or treat* or manag* or intervention*).tw. (7745138)
7. (mime* or miming or mirror* or tap* or massag* or stretch* or acupunctur* or needling* or biofeedback or neuromuscular* or kinesio* or cryo*).tw. (382977)
8. (electric* adj2 stimul*).tw. (62989)
9. (e-stim* or electromyograph* or semg).tw. (39537)
10. or/5-9 (8084566)
11. 4 and 10 (15595)
12. exp Central Nervous System Diseases/ (1342443)
13. (central nervous system adj2 (diseas* or injur* or infect*)).tw. (9406)
14. upper motor neuron.tw. (1424)
15. stroke*.tw. (218936)
16. brain injur*.tw. (57635)
17. tbi.tw. (21035)
18. (central adj3 (facial.tw. adj2 (paralys* or paresis or palsy or palsies))).tw. (144)
19. or/12-18 (1463675)
20. 11 and 19 (1970).
